# Decreased Expression of *ZNF554* in Gliomas is Associated with the Activation of Tumor Pathways and Shorter Patient Survival

**DOI:** 10.3390/ijms21165762

**Published:** 2020-08-11

**Authors:** Andrea Balogh, Lilla Reiniger, Szabolcs Hetey, Peter Kiraly, Eszter Toth, Katalin Karaszi, Kata Juhasz, Zsolt Gelencser, Agnes Zvara, Andras Szilagyi, Laszlo G. Puskas, Janos Matko, Zoltan Papp, Ilona Kovalszky, Csaba Juhasz, Nandor Gabor Than

**Affiliations:** 1 Systems Biology of Reproduction Research Group, Institute of Enzymology, Research Centre for Natural Sciences, H-1117 Budapest, Hungary; balogh.andrea@ttk.hu (A.B.); heteysz@gmail.com (S.H.); peter0kiraly@gmail.com (P.K.); toth.eszter@ttk.hu (E.T.); tika0604@gmail.com (K.K.); ho12ember@gmail.com (K.J.); gelzsolt@gmail.com (Z.G.); szilagyi.andras@ttk.hu (A.S.); 2 First Department of Pathology and Experimental Cancer Research, Semmelweis University, H-1085 Budapest, Hungary; reiniger.lilla@med.semmelweis-univ.hu (L.R.); koval@korb1.sote.hu (I.K.); 3 Laboratory of Functional Genomics, Department of Genetics, Biological Research Centre, H-6726 Szeged, Hungary; zvara.agnes@gmail.com (A.Z.); laszlo@avidinbiotech.com (L.G.P.); 4 Department of Immunology, Eotvos Lorand University, H-1117 Budapest, Hungary; matko@elte.hu; 5 Maternity Private Clinic, H-1126 Budapest, Hungary; pzorvosihetilap@maternity.hu; 6 Department of Obstetrics and Gynecology, Semmelweis University, H-1088 Budapest, Hungary; 7 Department of Pediatrics, Neurology, Neurosurgery, Wayne State University School of Medicine, Detroit, MI 48201, USA; csaba.juhasz@wayne.edu; 8 Barbara Ann Karmanos Cancer Institute, Detroit, MI 48201, USA

**Keywords:** glioma, glioblastoma, survival, transcriptome, zinc finger protein

## Abstract

Zinc finger protein 554 (ZNF554), a member of the Krüppel-associated box domain zinc finger protein subfamily, is predominantly expressed in the brain and placenta in humans. Recently, we unveiled that ZNF554 regulates trophoblast invasion during placentation and its decreased expression leads to the early pathogenesis of preeclampsia. Since ZNF proteins are immensely implicated in the development of several tumors including malignant tumors of the brain, here we explored the pathological role of ZNF554 in gliomas. We examined the expression of ZNF554 at mRNA and protein levels in normal brain and gliomas, and then we searched for genome-wide transcriptomic changes in U87 glioblastoma cells transiently overexpressing *ZNF554*. Immunohistochemistry of brain tissues in our cohort (*n* = 62) and analysis of large TCGA RNA-Seq data (*n* = 687) of control, oligodendroglioma, and astrocytoma tissues both revealed decreased expression of *ZNF554* towards higher glioma grades. Furthermore, low *ZNF554* expression was associated with shorter survival of grade III and IV astrocytoma patients. Overexpression of *ZNF554* in U87 cells resulted in differential expression, mostly downregulation of 899 genes. The “PI3K-Akt signaling pathway”, known to be activated during glioma development, was the most impacted among 116 dysregulated pathways. Most affected pathways were cancer-related and/or immune-related. Congruently, cell proliferation was decreased and cell cycle was arrested in *ZNF554*-transfected glioma cells. These data collectively suggest that ZNF554 is a potential tumor suppressor and its decreased expression may lead to the loss of oncogene suppression, activation of tumor pathways, and shorter survival of patients with malignant glioma.

## 1. Introduction

Zinc-finger proteins (ZNFs) belong to one of the largest transcription factor families in the human genome showing a great diversity of molecular functions [1]. Notably, in addition to DNA binding, studies have recently revealed the RNA, protein, and lipid interacting abilities of zinc finger motifs [2,3,4,5,6]. Nearly one-third of ZNFs belong to the Krüppel-associated box domain zinc finger protein (KRAB-ZNF) subfamily and contribute to transcriptional regulation during various cellular processes including differentiation, cell growth, and morphogenesis [7,8,9]. The majority of KRAB-ZNFs are involved in epigenetic silencing mediated by the KAP1 co-repressor via the induction of histone H3K9 trimethylation and DNA methylation [10,11].

ZNF554, a member of the KRAB-ZNF subfamily, is encoded in the KRAB-ZNF cluster on chromosome 19p13, which is highly coated by heterochromatin proteins and contains a large number of long interspersed nuclear elements (LINEs), denoting the tightly controlled regulation of its expression [12]. Surprisingly, our recent tissue qRT-PCR study revealed that *ZNF554* gene expression is mostly restricted to the brain and placenta in humans [13]. Furthermore, our genome-wide transcriptomic analysis showed the emerging role of ZNF554 as a hub transcription factor that drives deep trophoblast invasion, and the pathogenesis of preeclampsia is partly originated from its dysregulation. As functional evidence, *ZNF554*-silenced extravillous trophoblast cells had reduced migratory and invasive functions [13].

The remarkable *ZNF554* expression in the brain [13] was particularly interesting since ZNFs play a critical role in the development and differentiation of the nervous system. For instance, ZNF536 is abundant in the brain and is expressed in the developing central nervous system localized in the cerebral cortex, hippocampus, and hypothalamic area. ZNF536 expression is increased during cell differentiation and its overexpression inhibits retinoic-acid induced neuronal differentiation [14]. The importance of KRAB-ZNFs in the development and function of the brain is also supported by an interesting study, where differential expression of genes among human and chimpanzee tissues was compared. Nowick et al. found differential expression of 90 transcription factor genes only in the brain and revealed that they are organized in a co-expression network comprised of two modules, both enriched for primate-specific KRAB-ZNF genes [15].

Based on their essential role in transcription regulation, it is of major interest that many ZNF proteins act either as oncogenes or tumor suppressors [5,16], and are immensely implicated in the development of several tumors including gliomas [5]. Gliomas account for about 80% of all malignant primary brain tumors [17]. According to the most recent WHO classification criteria [18], most diffuse gliomas in adults belong to one of three molecular categories: Isocitrate dehydrogenase (IDH)-wildtype, IDH-mutant but 1p/19q-non-codeleted, or IDH-mutant and 1p/19q-codeleted. Histologically, 1p/19q-non-codeleted (IDH-wildtype or IDH-mutant) diffuse gliomas generally have an astrocytic phenotype, and the IDH-mutant and 1p/19q-codeleted tumors possess an oligodendroglial phenotype. Grade IV astrocytic glioma (glioblastoma multiforme (GBM)) is the most frequent adult glioma type and has the lowest survival rate [19]. Regarding the role of ZNFs in the development of gliomas, ZNF926 has been shown to promote cell proliferation and survival in gliomas [20], and stem cell maintenance in GBM [21]. In addition, Tatard et al. concluded in their study that antiproliferative functions of ZNF238 in normal granule neuron precursors and possibly other precursors counteract brain tumors (medulloblastoma and glioblastoma) formation [22].

Since ZNF554 is a regulator of cell invasion [13], and it has high expression in the brain, our collaborative teams [13,23,24] hypothesized that it may play a role in glioma development. Accordingly, we aimed to examine the expression of ZNF554 both at RNA and protein levels in normal brain and adult diffuse gliomas, as well as the association of *ZNF554* expression with patient survival, and to search for genome-wide transcriptomic and proteomic changes in glioblastoma cells transiently overexpressing *ZNF554*. To our knowledge, this is the first study that unveils the detailed expression pattern of ZNF554 in the human brain, the decreased expression of *ZNF554* towards higher tumor grades, and *ZNF554* as a favorable prognostic gene in adult diffuse gliomas. We also show that *ZNF554* overexpression in U87 glioblastoma cells leads to dysregulation of several pathways and genes known to be impacted in adult diffuse gliomas. These results suggest that *ZNF554* is a potential tumor suppressor and its decreased expression may lead to the activation of tumor pathways in adult diffuse gliomas, with a detrimental effect on patient survival.

## 2. Results

### 2.1. Brain Tissue Expression Pattern of ZNF554

Human tissue cDNA array expression profiling revealed that *ZNF554* mRNA expression is the highest in the brain amongst 47 human adult tissues included in the analysis [13]. In addition, the second to fourth top tissues (spinal cord, optic nerve, retina), which have ca. 60% lower *ZNF554* expression level than the brain, also belong to the nervous system (Supplementary Figure S1A). When we took a closer look into the brain expression of *ZNF554* using data from Allen Brain Atlas, and we found that *ZNF554* expression shows low region specificity. Only the white matter, striatum, and the epithalamus had significantly lower (*p* < 0.05 or *p* < 0.01) *ZNF554* expression than other brain regions (Supplementary Figure S1B).

### 2.2. ZNF554 Protein Expression in Gliomas

**Figure 1:**
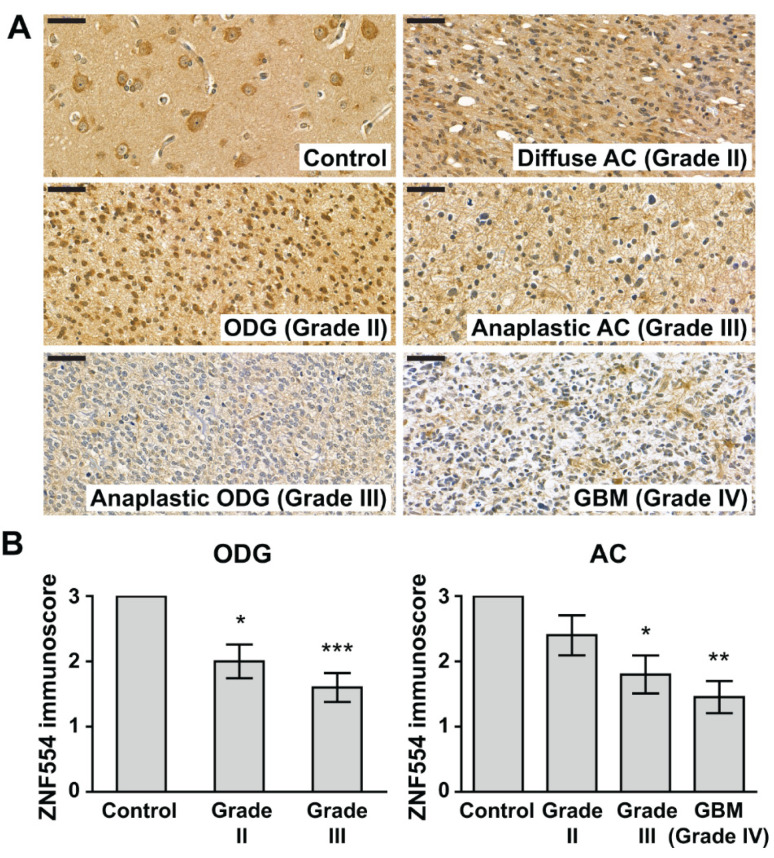
Zinc finger protein 554 (ZNF554) expression is decreased at the protein level in gliomas. (**A**) 5-µm-thick brain sections from controls (*n* = 11) and from patients with grade II (*n* = 10) or grade III (*n* = 10) oligodendroglioma, grade II (*n* = 10) or grade III (*n* = 10) astrocytoma, or glioblastoma (*n* = 11) were immunostained for ZNF554. ZNF554 protein abundance decreased with increasing tumor grades in patients with oligodendroglioma or with astrocytoma. Representative images, hematoxylin counterstain, 50 µm scale bar, 400× magnifications. (**B**) ZNF554 immunoscores (mean ± SEM) of patient groups are displayed on the left (control and oligodendrogliomas) and right (control and astrocytomas) graphs. Kruskal–Wallis with Dunn’s post hoc test was used for the statistical analysis (* *p* < 0.05, ** *p* < 0.01, *** *p* < 0.001). Astrocytoma, AC; glioblastoma, GBM; oligodendroglioma, ODG.

Immunohistochemistry (IHC) results showed that ZNF554 protein expression decreased with increasing tumor grades in patients with oligodendroglioma (ODG) and astrocytoma (AC). In detail, average ZNF554 immunoscores were 33% and 47% lower in grade II and grade III ODG tumors, respectively, than in controls (control: 3.00 ± 0.00; ODG grade II: 2.00 ± 0.26, *p* < 0.05; anaplastic ODG grade III: 1.60 ± 0.22, *p* < 0.001, Figure 1A,B). In ACs, the average ZNF554 immunoscores were 40% and 52% lower in grade III and grade IV tumors, respectively, than in controls (control: 3.00 ± 0.00; anaplastic AC grade III: 1.80 ± 0.29, *p* < 0.05; GBM grade IV: 1.45 ± 0.25, *p* < 0.01, Figure 1A,B). The mean immunoscore for ZNF554 was lower in grade II AC tumors as well, although the difference did not reach statistical significance (2.40 ± 0.31, Figure 1A,B).

### 2.3. ZNF554 mRNA Expression in Gliomas

**Figure 2:**
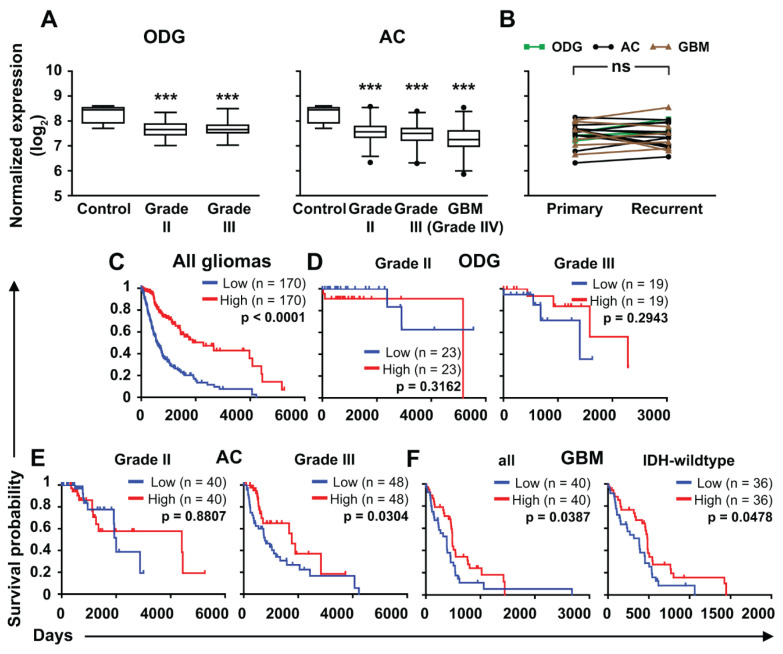
*ZNF554* mRNA expression is decreased in gliomas and positively associates with patient survival. (**A**) Box plots (median and whiskers: 1–99 percentile) represent normalized gene expression levels (log_2_ norm_count+1) of *ZNF554* from processed control brain (*n* = 5), ODG (grade II, *n* = 93; grade III, *n* = 75, **left graph**), AC (grade II, *n* = 161; grade III, *n* = 194), and GBM (grade IV, *n* = 159, **right graph**) RNA-Seq data from the TCGA database. One-way ANOVA with Dunnett’s post hoc test (*** *p* < 0.001) was used to compare glioma groups with the control group. (**B**) In 20 cases (*n*_ODG_ = 3, *n*_AC_ = 11, *n*_GBM_ = 6), *ZNF554* gene expression levels of primary and recurrent gliomas were compared by paired *t*-test and depicted on a before-after plot graph (ns: *p* > 0.05). Outcome predictions of all gliomas (*n* = 682, **C**), ODG (grade II, (**D**) **left graph**), anaplastic ODG (grade III, (**D**) **right graph**), diffuse AC (grade II, (**E**) **left graph**), anaplastic AC (grade III, (**E**) **right graph**), all (IDH-wildtype and IDH-mutant) GBM (grade IV, (**F**) **left graph**), and IDH-wildtype GBM (grade IV, (**F**) **right graph**) cases based on TCGA overall survival data were linked to *ZNF554* mRNA expression level. Kaplan–Meier plots show survival correlations with high (red line) or low (blue line) expression levels of *ZNF554*. The lower and upper quartiles of patients were analyzed by the Log-rank (Mantel-Cox) test (*p* < 0.05 was considered significant). Astrocytoma, AC; glioblastoma, GBM; isocitrate dehydrogenase, IDH; oligodendroglioma, ODG.

Next, we aimed to confirm our findings at the mRNA level on larger cohorts. Therefore, we analyzed *ZNF554* mRNA expression in gliomas from the TCGA database [25]. We found that the *ZNF554* log_2_ expression level was lower in all glioma groups (ODG grade II: 7.673 ± 0.027, ODG grade III: 7.659 ± 0.030, AC grade II: 7.579 ± 0.029, AC grade III: 7.458 ± 0.027, GBM: 7.241 ± 0.035, *p* < 0.001 in all cases) compared to controls (8.272 ± 0.161, Figure 2A). Of note, there was no difference in *ZNF554* expression in primary and recurrent gliomas Figure 2B). Furthermore, the survival probability of patients with high *ZNF554* expression was higher than those with low *ZNF554* expression when all gliomas were analyzed together (*p* < 0.001, Figure 2C). When gliomas were analyzed based on their type and grade, low *ZNF554* expression associated with shorter survival of anaplastic AC (grade III, *p* < 0.05) and GBM (grade IV, *p* < 0.05) patients (including a subgroup of IDH-wildtype GBMs, *p* < 0.05), but not with the survival of patients of the two ODG groups and the grade II AC group (Figure 2D–F).

### 2.4. The Effect of ZNF554 Overexpression in Glioblastoma Cells

**Figure 3:**
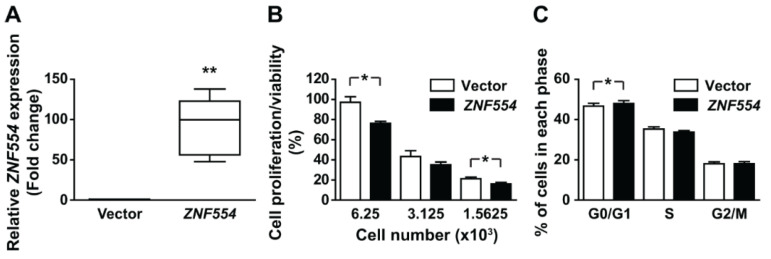
Overexpression of *ZNF554* decreases cell proliferation and induces cell cycle arrest in U87 glioblastoma cells. (**A**) Box plots (median and whiskers: 1–99 percentile) represent the relative gene expression level of *ZNF554* in U87 cells two days after transient transfection with pCMV-ZNF554 or empty vector. Quantitative RT-PCR data are from five independent experiments. Unpaired *t*-test with Welch correction was used for the statistical analysis (** *p* < 0.01). (**B**) Analysis of cell proliferation/viability of vector- and *ZNF554*-transfected U87 cells by the Cell Counting Kit-8 assay. The percentage of cell proliferation relative to the vector-transfected control cells are presented as the mean ± SEM. (**C**) Analysis of cell cycle distribution of vector- and *ZNF554*-transfected U87 cells by Hoechst33342 staining and flow cytometry. Percentage of cells (mean ± SEM) in G0/G1, S, and G2/M phases are shown. Data were retrieved from three-four independent experiments. Paired *t*-test was used for the statistical analysis (* *p* < 0.05).

To reveal the impact of ZNF554 expression on glioblastoma cells, we transiently overexpressed *ZNF554* in U87 glioblastoma cells. There was an average 92-fold increase in *ZNF554* expression in transfected cells compared to cells transfected with the empty vector (** *p* < 0.01, Figure 3A).

To further determine whether *ZNF554* is a potential tumor suppressor in gliomas, the effect of *ZNF554* on proliferation/viability and cell cycle of U87 cells was examined. After transient transfection with *ZNF554*, cell proliferation/viability decreased (*p* < 0.05, Figure 3B), and the number of U87 cells in the G0/G1 phase slightly but significantly increased from 46.65% to 48.05% (*p* < 0.05, Figure 3C). These results suggested that cell proliferation inhibition by *ZNF554* is likely mediated through cell cycle arrest at G0/G1.

**Figure 4:**
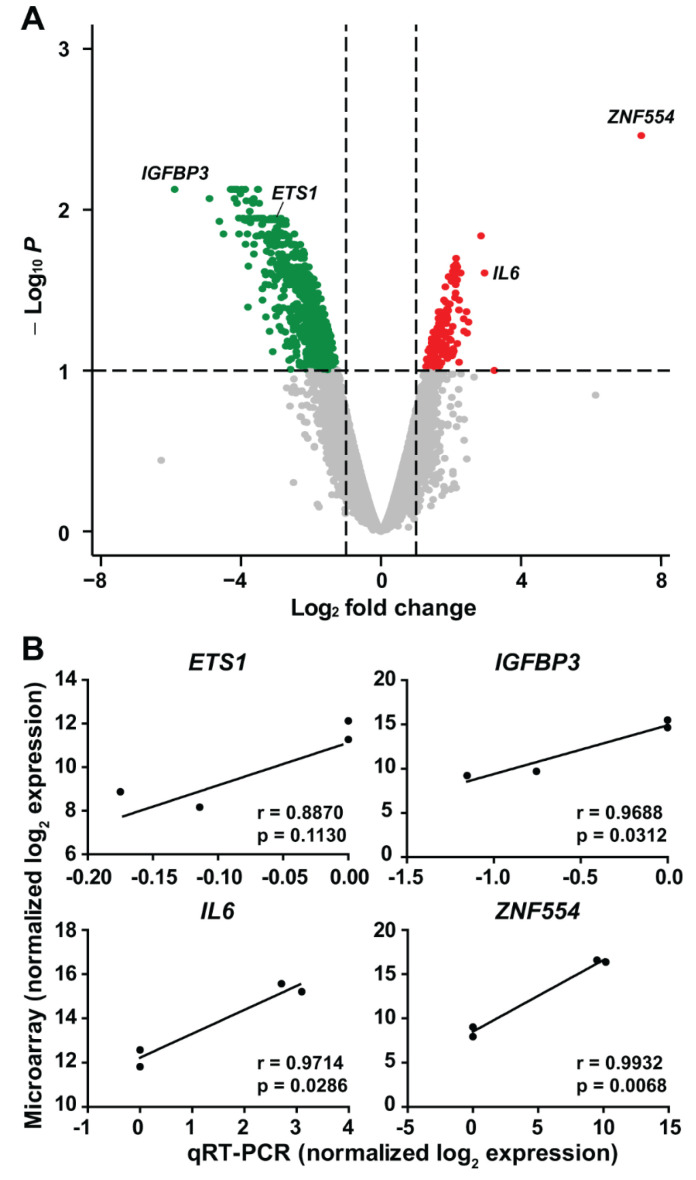
Genome-wide expression changes in U87 glioblastoma cells overexpressing *ZNF554*. (**A**) All 18,834 genes are represented in terms of their measured differences in abundance (*x*-axis) and the significance of the difference (*y*-axis) on a volcano plot. The significance is represented as a negative log (base 10) of the adjusted *p*-value so that more significant differences in expression are plotted higher on the *y*-axis. Dotted lines represent the thresholds used to select the probes for differentially expressed (DE) genes: <−1 and >1 for the magnitude of differential expression and pFDR of <0.1 for statistical significance. According to these criteria, of the 899 DE genes, 145 were up-regulated (depicted with red), while 754 were down-regulated (depicted with dark green) in U87 cells transfected with *ZNF554*. (**B**) Correlation of microarray and qRT-PCR results for the selected genes (*ETS1*, *IGFBP3*, *IL6*, and *ZNF554*). The differences in the transcription of genes were log_2_-transformed and plotted against each other. Statistical results of Pearson’s correlation are indicated (*p* < 0.05 was considered significant). Two independent experiments in duplicates were performed. ETS proto-oncogene 1, ETS1; insulin-like growth factor binding protein 3, IGFBP3; interleukin 6, IL6; positive false-discovery rate, pFDR; zinc finger protein 554, ZNF554.

Genome-scale gene expression analysis identified dysregulation of 4.77% (899/18 834) of genes expressed in U87 glioblastoma cells overexpressing *ZNF554* (Supplementary Table S1, Figure 4A). Of importance, the vast majority (84%, 754/899) of these genes were downregulated, which is consistent with the overall repressive role of KRAB-ZNFs on gene transcription [8]. Among the top 20 upregulated genes were several cytokines (*IL6*, interleukin 6; *IL18*, *IL36B*) and chemokines (*CCL20*, C-C motif chemokine ligand 20; *CCL5*, *CXCL2*, C-X-C motif chemokine ligand 2), as well as *CDA* (cytidine deaminase), *CCDC134* (coiled-coil domain containing 134), and two HLA class II major histocompatibility antigens (*HLA-DRB3*, *HLA-DRB5*). Among the top 20 downregulated genes were a chemokine (*CCL8*), an integrin (*ITGB1*), two UDP-glucuronosyltransferases (*UGT1A6*, *UGT1A8*), transcripts for several proteins found in the endoplasmic reticulum (*SERP1*, stress associated endoplasmic reticulum protein 1; *SEC23A*, Sec23 homolog A, COPII coat complex component; *TMED2*, transmembrane p24 trafficking protein 2), a proto-oncogene (*CASC4*, cancer susceptibility 4) and *IGFBP3* (insulin-like growth factor-binding protein 3). Of note, in the case of four genes selected for validation, there was a good correlation between microarray and qRT-PCR results (Figure 4B).

**Figure 5:**
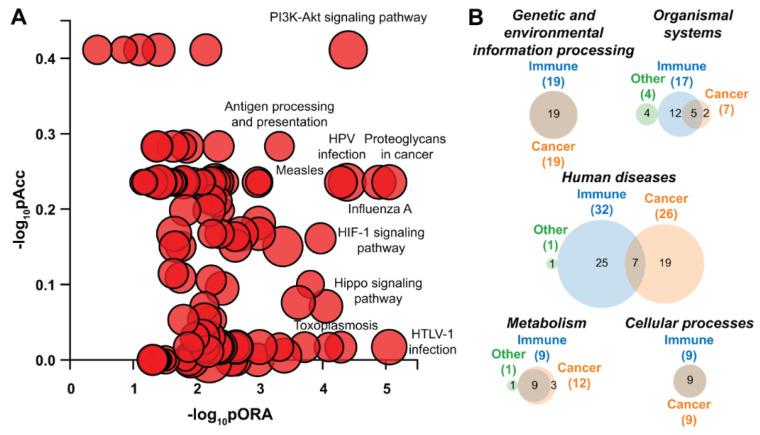
Pathways perturbation vs. over-representation and Venn diagrams in U87 glioblastoma cells overexpressing *ZNF554*. (**A**) Pathways (the 10 most dysregulated ones are indicated) were plotted according to two types of evidence computed by iPathwayGuide: Over-representation on the *x*-axis (pORA) and the total pathway accumulation on the *y*-axis (pAcc). For both, measured *p*-values are displayed on the negative log (base 10) scale. (**B**) Dysregulated pathways in U87 cells transfected with *ZNF554* were categorized in five Kyoto Encyclopedia of Genes and Genomes (KEGG) pathway subgroups. Venn diagrams show the overlap between immune- and cancer-related biological pathways in these subgroups. Akt serine/threonine kinase, Akt; hypoxia-inducible factor 1-alpha, HIF-1; human papillomavirus, HPV; human T-cell leukemia virus 1, HTLV-1; phosphatidylinositol 3-kinase, PI3K.

**Figure 6:**
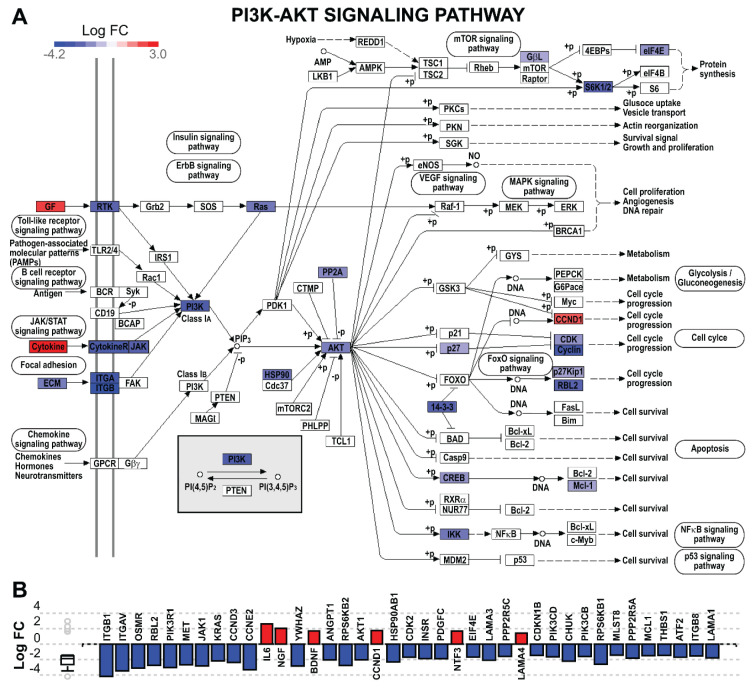
The PI3K-Akt signaling pathway is downregulated in U87 glioblastoma cells overexpressing *ZNF554*. (**A**) The Kyoto Encyclopedia of Genes and Genomes (KEGG) pathway diagram (KEGG: 04151) is overlaid with the computed log fold change of each gene. The downregulation is shown in dark blue, and the upregulation in dark red. The legend describes values on the gradient. (**B**) Gene log fold change bar plot. All genes in the PI3K-AKT signaling pathway were ranked according to log fold change, negative values were depicted in blue and positive values in red. The box and whisker plot on the left summarizes the distribution of all gene perturbations in this pathway, the box represents the first quartile, median and third quartile, while circles represent outliers. Akt serine/threonine kinase, Akt; phosphatidylinositol 3-kinase, PI3K.

Analysis with iPathwayGuide to investigate biological processes and pathways dysregulated in U87 glioblastoma cells overexpressing *ZNF554* revealed that the most impacted Kyoto Encyclopedia of Genes and Genomes (KEGG) pathways included *“PI3K-Akt signaling”* and *“proteoglycans in cancer”*, both cancer-related pathways (Supplementary Table S2, Figure 5A and Figure 6). Among the 116 dysregulated pathways, 73 were cancer-related, 86 were immune-related, and 49 pathways were common (Supplementary Table S2, Figure 5B).

Among 633 gene ontology (GO) biological processes dysregulated after positive false-discovery rate (pFDR) correction, the most impacted were *“phosphorus metabolic process”*, *“phosphate-containing compound metabolic process”*, *“cellular response to chemical stimulus”*, and *“vesicle-mediated transport”* (Supplementary Table S3). We found the most impacted GO molecular functions to be *“protein binding”*, *“carbohydrate derivative binding”*, *“anion binding”*, and *“nucleotide binding”* among 68 dysregulated molecular functions (Supplementary Table S4).

## 3. Discussion

### 3.1. Principal Findings of This Study:

(1) *ZNF554* shows predominant brain expression with low region specificity in humans; (2) in adult diffuse gliomas, *ZNF554* mRNA and protein expressions decrease with increasing tumor grade; (3) low *ZNF554* mRNA expression is associated with shorter survival of anaplastic astrocytoma and glioblastoma patients, and also when all gliomas are analyzed together; (4) the overexpression of *ZNF554* results in the dysregulation of 899 genes in U87 glioblastoma cells, and most of these are downregulated; (5) among 116 dysregulated pathways, 73 are cancer-related and 86 are immune-related (49 pathways are common), and these are mostly downregulated; and (6) the overexpression of *ZNF554* decreases cell proliferation and induces cell cycle arrest.

### 3.2. ZNFs in the Normal Human Brain

The largest TF family in mammals is the family of KRAB-ZNF genes, and about one-third of these genes are primate-specific and evolved by recent gene duplications [26]. The rapid evolution of these genes in the human lineage is also demonstrated by the fact that there is an enrichment of KRAB-ZNF genes among DE genes in the human brain compared to chimpanzees [15]. Moreover, among the ~350 KRAB-ZNFs expressed in humans, a larger number of KRAB-ZNFs are expressed in the brain than in most other adult human tissues [9,27]. This evidence collectively suggests key roles of KRAB-ZNFs in brain development and the expansion of higher brain functions in humans. Of note, chromosome 19 contains the most KRAB-ZNF gene clusters, and *ZNF554* is a member of the chr19p13.3 gene cluster [28]. Our results showed that *ZNF554* has predominant brain expression among adult tissues with low region specificity. In a recent study, approximately 150 gene promoters were counted as binding sites for ZNF554 in the brain, and its expression is constant in space and time during neurodevelopment [29]. Besides ZNF554, Farmiloe et al. found that 50 other KRAB-ZNFs with more than 50 binding sites on gene promoter regions are expressed in the human brain, and these TFs can regulate gene expression in a region-specific manner during human brain development [29].

### 3.3. ZNFs are Double-Edged Swords in Different Cancer Types

We revealed decreased *ZNF554* expression in adult diffuse gliomas with increasing tumor grade at both protein and mRNA levels, which possibly reflects its tumor suppressor nature in gliomas. Furthermore, lower *ZNF554* expression was associated with worse prognosis in grade III ACs and GBMs. Those patients having the worst survival outcomes (IDH-wildtype GBM) showed longer overall survival with high *ZNF554* expression. Interestingly, we found no apparent survival advantage of high *ZNF554* expression for ODGs or low-grade ACs. The reason for these differences remains to be determined, but the lack of a significant survival effect for ODGs may be explained, at least partly, by the lower case numbers. Still, as Figure 2D demonstrates, high expressors in the Grade III ODG group actually lived longer than the low expressors, and this difference may well become significant in a larger cohort. For Grade II AC, the case numbers do not explain the lack of survival differences; although there were a few very long survivors (>10 years) that were from the high expressor group.

*ZNF671*, also located on Chromosome 19, might play a tumor suppressor role in cancer since it was found downregulated in eight cancer-related *ZNF671* single-cell RNA-Seq datasets. The heterogeneous functional states of cell subgroups and correlation analysis showed that *ZNF671* played tumor suppressor roles in heterogeneous cancer cell populations. Western blot and transwell assays identified that *ZNF671* inhibited epithelial-mesenchymal transition, migration, and invasion of central nervous system cancers, lung cancer, melanoma, and breast carcinoma in vitro [30]. Interestingly, a recent analysis of TCGA GBM samples [31] revealed that only 1.5% (3/201) of genes associated with unfavorable prognosis in GBM are ZNF genes, while 18% (12/67) of genes associated with favorable prognosis in GBM are ZNF genes. However, (KRAB-)ZNFs may be double-edged swords since they could act as both tumor suppressors or oncogenes [5,32,33]. Even one ZNF can play different roles in different cancer types and upon various stimuli. For example, *ZNF395* expression is induced under hypoxic stress in GBM, where it up-regulates interferon-stimulated and cancer-related genes, promoting inflammation and cancer progression [34]. In contrast, the tumor suppressor role of *ZNF395* was proven in liver cancer, since the overexpression of miR-525-3p downregulated *ZNF395*, which promoted cancer cell migration and invasion [35]. These studies collectively showed that ZNFs may play different roles in different cancer types.

### 3.4. ZNF554 as a Potential Tumor Suppressor in Gliomas

To functionally investigate the potential tumor suppressor role of *ZNF554* in gliomas, we performed genome-wide expression profiling of U87 glioblastoma cells transiently overexpressing *ZNF554* and found that *ZNF554* overexpression induced the upregulation of 145 genes and the downregulation of 754 genes. Functional analysis of these DE genes demonstrated a close correlation with cancer- and immune-related pathways. The *“PI3K-Akt signaling”* was the most significantly enriched pathway, and genes involved in this process included *AKT1*, *JAK1*, *KRAS*, *MET*, and *PIK3R1*. The PI3K-Akt signaling pathway regulates various cellular processes such as cell cycle, apoptosis, and cytoskeletal rearrangement [36]. This pathway can be activated by various growth factors, cytokines, chemokines, hormones, extracellular matrix components, etc., through their respective receptors (e.g., RTKs, cytokine receptors, GPCRs, adhesion molecules). It is located upstream to other pathways involved in cancerogenesis (e.g., mTor, Ras, MAPK, FoxO, and insulin signaling pathways) [37,38,39,40,41,42] which were also dysregulated in *ZNF554*-transfected cells. The importance of the PI3K-Akt signaling pathway in glioma is proven by ongoing clinical trials in brain tumors targeting, amongst others, PI3K and Akt [43,44]. Furthermore, we found decreased proliferation and cell cycle arrest of *ZNF554*-transfected cells, which might be due to the downregulation of several cyclin-dependent kinases (*CDK2*, *CDK9*, *CDK10*, *CDK13*, *CDK17*), and five (*CCNC*, *CCND3*, *CCNE2*, *CCNG1*, *CCNG2*) out of six cyclin genes. This is in strong concordance with recent studies, one showing the anti-glioma effect of CDK inhibitors already in preclinical/clinical use [45], the other finding several CDKs and cyclin genes amongst top hub nodes in glioma network analysis [46].

Similar to our study, the enrichment of “allograft rejection”, “systemic lupus erythematosus”, “graft-versus-host disease”, “type I diabetes mellitus”, “antigen processing and presentation”, and “autoimmune thyroid disease” immune-related pathways were also found by Wang et al. analyzing DE genes in glioblastoma [47]. It was suggested by others that enhanced HLA class II expression in tumor cells may have significance in tumor immunogenicity, probably through enhanced direct tumor cell killing [48]. In addition, downregulation of major HLA class I and II genes in migrating cells in vitro and in invading cells in vivo was found in a glioma study [49].

### 3.5. Potential Diagnostic and Therapeutic Implications of ZNF554 in Gliomas

Our results suggest that *ZNF554* may be a novel prognostic biomarker for clinical patient management, especially in those with grade III and IV gliomas. Future studies should evaluate if low-grade gliomas lose *ZNF554* as they undergo malignant transformation into high-grade gliomas. Our in vitro studies suggest that enhanced expression of *ZNF554* in such high-grade gliomas has the potential to suppress tumor growth and invasiveness. Thus, the potential promotion of its expression by gene therapy to restore ZNF554’s tumor suppressor effect may provide therapeutic advantages and improve patient outcomes in adult diffuse gliomas. Such an effect would be particularly impactful in patients with the worst prognosis and severe treatment resistance with the current therapeutic options, such as those with IDH-wildtype GBM, where higher *ZNF554* expression appeared to be beneficial for survival. Delivery vectors such as viral vectors, liposomes, non-polymeric and polymeric nanoparticles have been used in gene therapy of GBM [50]. For example, a Phase I clinical trial started in 1999 for the treatment of recurrent malignant glioma with adenovirus-mediated p53 TF gene therapy [51]. However, later it was suggested to be effective as a supplementary therapy because of insufficient gene transfer, lack of bystander effect, and tolerance arising from genetic heterogeneity of glioma [52,53]. Nevertheless, TFs have great potential as targets in the individualized therapeutic approaches to cancer [54]. Moreover, a combination of various therapies, e.g., gene-and chemotherapy, gene-and immunotherapy may further improve the effectiveness of glioma treatment.

### 3.6. Strengths and Limitations

The strengths of our study are as follows: (1) Strict clinical definitions and homogenous patient groups; (2) the use of leading bioinformatics tools for microarray, gene ontology, and pathway analyses; and (3) the immunohistochemical evaluation of a clinically and histologically well-characterized human diffuse glioma collection.

Limitations of our study are as follows: (1) Due to the strict selection criteria, we could include a relatively modest number of cases into the analysis of survival data, especially in some of the glioma subgroups; (2) the number of samples was modest in the microarray and IHC experiments; nevertheless, microarray results are meaningful and fit well with what was known about glioma molecular development, while IHC results corroborate our findings on the large TCGA data set showing decreased expression of *ZNF554* at the mRNA level as well; and (3) we could only use a glioblastoma cell line and not primary glioblastoma cells for the overexpression of *ZNF554* since we had no tissue source for primary cells. While U87 cells used in our study are among the most commonly used glioma cell lines in neuro-oncology research, future studies could involve other glioma cell lines to test baseline expression of ZNF554 and its expression in overexpression/siRNA systems.

## 4. Conclusions

*ZNF554* is a potential tumor suppressor, and its decreased expression may lead to the loss of oncogene suppression, activation of tumor pathways, and shorter survival in malignant glioma patients. Although in vitro experiments were performed on an astrocytic glioma cell line, TCGA *ZNF554* mRNA expression data indicate the importance of ZNF554 in the pathophysiology of oligodendrogliomas as well. *ZNF554* overexpression in U87 glioblastoma cells induced gene expression changes associated with cancer-and immune-related pathways, with the PI3K-Akt signaling pathway enriched most significantly. Further studies, including single-cell RNA-seq analysis (with deep sequencing) on normal and tumorous human brain, are required to understand the precise role of *ZNF554* in brain functions and oligodendroglial as well as astrocytic glioma development, and to reveal its diagnostic and therapeutic potential in the prognostication and targeted therapy of adult diffuse glioma patients.

## 5. Materials and Methods

### 5.1. Analysis of ZNF554 mRNA Expression in Normal Brain

Microarray data (Allen Human Brain Atlas data 2010 [55]) from six donors were downloaded from http://human.brain-map.org in log_2_ format. The 169 different brain regions were grouped in 23 structural regions and *ZNF554* expression levels (probe set ID: A_23_P343250) were visualized on a bar chart.

### 5.2. Analysis of ZNF554 Protein Expression in Gliomas

Formalin-fixed paraffin-embedded (FFPE) brain biopsy specimens of 62 patients were studied from the archives of the First Department of Pathology and Experimental Cancer Research, Semmelweis University. Tumor tissues were classified according to the WHO classification of Tumors of the Central Nervous System [18]. Based on the presence/absence of histological features, diffuse gliomas, within oligodendroglial or astrocytic phenotype, are graded as WHO grade II (low-grade), WHO grade III (anaplastic), or WHO grade IV (glioblastoma), the latter two referred to as high-grade gliomas [56]. Permissions to use the archived tissues were obtained from the Local Ethics Committee (TUKEB Approval No.: 155/2012) and the study was conducted in accordance with the Declaration of Helsinki.

Fifty-one brain tumor tissues, including 10 cases of each diffuse astrocytoma (AC), anaplastic AC, oligodendroglioma (ODG), anaplastic ODG, as well as 11 cases of GBM were selected for the immunostaining. In addition, 11 samples of normal brain tissue, confirmed by histopathology, from patients who underwent surgery for epilepsy were used as controls. Clinical information for these patients is included in Table 1.

### 5.3. Immunohistochemistry for ZNF554

Five-micrometer-thick tissue sections were cut and mounted on SuperFrost^TM^ Plus slides (Thermo Fisher Scientific, Waltham, MA, USA) and stored at 4 °C until the staining. Immunostaining was carried out using the Novolink Polymer Detection System (Leica-Novocastra, Nussloch, Germany) according to the manufacturer’s protocol. Slides were dewaxed using xylene and rehydrated in graded alcohol series. Endogenous peroxidases were blocked with 10%H_2_O_2_ in methanol (20 min, room temperature). Antigen retrieval was performed at 37 °C for 5 min using the Bond Enzyme Pretreatment Kit (Leica Biosystems). After blocking non-specific binding with the Novocastra Protein Block (10 min, room temperature), slides were incubated with mouse polyclonal anti-ZNF554 antibody (Abnova, Taipei, Taiwan) at 1:50 dilution overnight at 4 °C. After post-primary amplification (30 min, room temperature), slides were incubated with the Novolink Polymer (30 min, room temperature) and visualized with 3,3′-diaminobenzidine (DAB) for 10 min. Finally, sections were counterstained with hematoxylin, and these were mounted (DPX Mountant; Sigma-Aldrich, St. Louis, MO, USA) after dehydration. The primary antibody was omitted in negative controls.

### 5.4. Evaluation of ZNF554 Immunostainings

Visual evaluation of ZNF554 immunostainings was performed microscopically (Carl Zeiss Microscopy GmbH, Jena, Germany) by a pathologist (LR) blinded to the clinical data. The intensity of the staining was recorded as absent, weak, moderate, or strong. The percentage of positive tumor cells was scaled as follows: (i) Less than 10%, (ii) 10–50%, and (iii) more than 50% positive tumor cells. ZNF554 protein expression was defined negative/weak (score 1) if less than 10% of the cells showed any positivity; medium (score 2) if 10–50% of the cells showed any positivity or more than 50% of the cells showed weak/moderate positivity; and high (score 3) if more than 50% of the cells showed strong positivity.

### 5.5. Analysis of ZNF554 mRNA Expression and Patients’ Survival in Gliomas

Based on The Cancer Genome Atlas (TCGA) Lower-Grade Glioma and Glioblastoma Multiforme study [57], normal brain, ODG (grades II and III), AC (grades II and III), and GBM (grade IV) *ZNF554* RNA-Seq data were downloaded from the UCSC Xena platform [58] (https://xenabrowser.net/) in log_2_ normalized form (log_2_ norm_count+1). Data were filtered for patients with known *ZNF554* expression and survival data. Patients were reclassified according to the latest WHO glioma classification criteria [18], based on molecular data. Finally, 687 patients (Supplementary Table S5) were included in the analysis: normal brain (*n* = 5), ODG (grade II, *n* = 93), anaplastic ODG (grade III, *n* = 75), diffuse AC (grade II, *n* = 161), anaplastic AC (grade III, *n* = 194), and GBM (grade IV, *n* = 159). *ZNF554* expression levels in different patient groups or primary and recurrent glioma cases (*n* = 20) were visualized on box plots and before-after plots, respectively, using the GraphPad Prism 5 software.

Outcome predictions of all gliomas together, as well as grade II ODG, grade III ODG, grade II AC, grade III AC, and grade IV GBM cases based on TCGA survival data linked to *ZNF554* mRNA expression level, were visualized on Kaplan–Meier plots with high or low. In the analysis, patients with *ZNF554* expression in the lower and upper quartiles were included.

### 5.6. Transient Transfection of U87 Glioblastoma Cells with ZNF554

Human U87 glioblastoma cells, obtained from the American Type Culture Collection (ATCC, Manassas, VI, USA), were cultured in Dulbecco’s modified Eagle’s medium (DMEM; Gibco-Thermo Fisher Scientific) supplemented with 10% fetal bovine serum (FBS; Gibco-Thermo Fisher) at 37 °C in a humidified 95% air and 5% CO_2_ atmosphere. Cells were transfected with expression plasmid pCMV6-ZNF554 (OriGene, Rockville, MD, USA) or empty vector pCMV6 (OriGene) using Lipofectamine-2000 (Thermo Fisher Scientific) following the manufacturer’s instructions. Transfection media was replaced with fresh medium six hours post-transfection. Cells were split into two batches 24 h after transfection, harvested 48 h after transfection, washed twice with ice-cold Dulbecco’s PBS, and then pellets were stored at −80 °C until use.

### 5.7. Total RNA Isolation

Total RNA was isolated from U87 cells by the Direct-zol^TM^ RNA MiniPrep kit (Zymo Research, Irvine, CA, USA) according to the manufacturers’ protocols. RNA concentrations were measured on a NanoDrop ND-1000 Spectrophotometer (Thermo Fisher Scientific). RNA integrity and quality were assessed on a Bioanalyzer 2100 (Agilent Technologies, Santa Clara, CA, USA). Isolated RNA was stored at −80 °C until use.

### 5.8. Labeling and Microarray Hybridization

One microgram of total RNA was first reverse transcribed in 10 μL volume using Oligo(dT) Primer and ArrayScript^TM^ enzyme (Thermo Fisher Scientific), then the second cDNA strand was synthesized in 50 μL final volume using DNA Polymerase and RNase H (Thermo Fisher Scientific). Amino allyl-modified antisense RNA (aaRNA) was then synthesized by in vitro transcription using amino allyl-modified UTP and T7 Enzyme mix. All these steps were done using the Amino Allyl MessageAmp^TM^ II aRNA Amplification Kit (Ambion-Thermo Fisher Scientific), according to the manufacturer’s instructions. Six μg of amino allyl-modified amplified RNA was labeled with Cy3 dye in 10 μL volume according to the manufacturer’s instructions (One-Color Quick Amp Labeling Kit, Agilent Technologies) and purified with RNeasy Mini Kit (Qiagen). The incorporation rate of the dye and the labeled aaRNA concentration were detected using NanoDrop 3.1.0. The incorporation rate of the samples was 30–60 dye molecules per 1000 nucleotides.

The human oligonucleotide microarray (Whole Human Genome Microarray, 4 × 44 K, G2519F, Design ID 014850, Agilent Technologies) was used to determine gene expression changes. 825 ng of Cy3 labeled aaRNA, 11 μL 10X Blocking Agent and 2.2 μL 25X Fragmentation Buffer were mixed in a final volume of 55 μL and fragmented at 60 °C for 30 min then 55 μL 2X GEx Hybridization Buffer were added to each sample to stop the fragmentation reaction. All these steps were done using the Gene Expression Hybridization Kit of Agilent Technologies according to the manufacturer’s instructions. A hundred microliters of these mixes were used to fill the 4-plex backing slides and the microarray was placed onto it. This “hybridization sandwich” was assembled in an Agilent microarray hybridization chambers. The chambers were then loaded into a hybridization rotator rack (~5 rpm) and incubated at 65 °C for 17 h. After hybridization, the slides were washed in Wash buffer 1 at room temperature for 1 min then in Wash buffer 2 at 37 °C for another 1 min before scanning. Each array was scanned at 543 nm (for Cy3 labeling) in an Agilent Scanner (G2505B) using the extended dynamic range function with 5μm resolution. Output image analysis and feature extraction were done using Feature Extraction 9.5.1 software using the single-color gene expression protocol (GE1_1100_Jul11) [59].

### 5.9. Quantitative Real-Time PCR

Five hundred nanograms of total RNA was transcribed by the qScript cDNA Synthesis kit (Quantabio, Beverly, MA, USA). TaqMan^TM^ Gene Expression Assays (Table 2) and Universal PCR Master Mix (Applied Biosystems-Thermo Fisher Scientific) were used for gene expression quantification on a Biomark system (Fluidigm, San Francisco, CA, USA). Ct values for each target gene (*ETS1*, *IGFBP3*, *IL6*, and *ZNF554*) and the endogenous control (*RPLP0*) were averaged over three technical replicates. For each target gene, the fold change was calculated using the delta-delta Ct method. In the correlation analysis, fold change data were log_2_ transformed.

### 5.10. Proliferation/Viability Assay

Forty-eight hours after transfection, the Cell Counting Kit-8 (Merck-Sigma Aldrich) colorimetric assay was used to measure cell proliferation, according to the manufacturer’s instruction. The mean optical density (OD at 450 nm) of three wells for the groups was used to calculate the percentage of cell proliferation relative to the vector-transfected control cells.

### 5.11. Cell Cycle Assay

Forty-eight hours after transfection, 2.5 μg/mL Hoechst33342 (Thermo Fisher Scientific) was added to the cells. Samples were incubated at 37 °C for 1 h then were trypsinized and measured on a Cytoflex instrument (Beckman Coulter, Brea, CA, USA). Twenty thousand events were collected per sample. Late apoptotic/necrotic cells, stained by propidium iodide (Merck-Sigma Aldrich), were excluded from the analysis.

### 5.12. Data Analysis

#### 5.12.1. Allen Brain Atlas Data Analysis

Downloaded *ZNF554* mRNA expression data were averaged to larger brain regions and statistical analysis was performed by Kruskal–Wallis with Dunn’s post hoc test to compare the groups. *p*-values of <0.05 were considered significant.

#### 5.12.2. TCGA RNA-Seq Data Analysis

Downloaded data were filtered for *ZNF554* expression in the normal brain and glioma tissues. Mixed gliomas were excluded from the analysis. One-way ANOVA with Dunnett’s post hoc test was used to compare glioma groups with the control group for *ZNF554* expression. The paired *t*-test was used to compare *ZNF554* expression in primary and recurrent gliomas. The Log-rank (Mantel–Cox) test was used for survival analysis. *p*-values of <0.05 were considered significant.

#### 5.12.3. Microarray Data Analysis of Transfected U87 Cells

For U87 cells overexpressing *ZNF554*, analyses were performed in *R* statistical environment using the limma package of Bioconductor (www.bioconductor.org). Following quality control performed by arrayQualityMetrics [60], expression data were background corrected with the normexp method and quantile normalized. Prior to differential gene expression analysis, irregular replicate probes were averaged with avereps function of the limma package. Genes were selected as differentially expressed upon a False Discovery Rate (FDR) [61] adjusted *p*-value of< 0.1 and fold-change of ≥2. The hgug4112a.db Agilent whole-genome database was used for gene annotation.

#### 5.12.4. Pathway Analysis

To identify significantly impacted pathways, biological processes, and molecular functions, we compared gene expression between U87 cells transfected with an empty vector or with *ZNF554* using Advaita Bio’s iPathwayGuide (https://www.advaitabio.com/ipathwayguide). This software analysis tool implements the ‘Impact Analysis’ approach that takes into consideration the direction and type of all signals on a pathway, the position, role, and type of every gene [62].

#### 5.12.5. Quantitative RT-PCR Data Analysis

Unpaired *t*-test with Welch correction was used to compare the relative expression of *ZNF554* between cells transfected with pCMV-ZNF554 vs. empty vector from five independent experiments. Pearson’s correlation analysis was used to calculate the correlation strength and direction between microarray and qRT-PCR data for the selected genes from two independent experiments. *p*-values of <0.05 were considered significant.

#### 5.12.6. Data Analysis of ZNF554 Immunostainings

Kruskal–Wallis with Dunn’s post hoc test was used to compare mean immunoscores between control and patient groups. *p*-values of <0.05 were considered significant.

#### 5.12.7. Data Analysis of Cell Proliferation/Viability and Cell Cycle

The paired *t*-test was used to compare proliferation (background-corrected optical density at 450 nm) or cell cycle status (percent of cells in G0/G1, S, and G2/M phases) of cells transfected with pCMV-ZNF554 or empty vector from three-four independent experiments. *p*-values of <0.05 were considered significant.

## Figures and Tables

**Table 1:** Demographic and clinical characteristics of the immunohistochemistry study groups.

Groups	Grade	*n* ^a^	Age (Years) at Diagnosis ^a^	Male/Female ^b^
Control (epilepsy)	-	11	38.0 (14–64)	5/6
Oligodendroglioma	II	10	51.5 (29–65)	3/7
Anaplastic oligondendroglioma	III	10	47.0 (30–67)	3/7
Diffuse astrocytoma	II	10	47.0 (29–69)	4/6
Anaplastic astrocytoma	III	10	41.5 (31–78)	6/4
Glioblastoma multiforme	IV	11	64.0 (24–73)	6/5

^a^ Values are presented as numbers. ^b^ Values are presented as median (interquartile (IQR) range).

**Table 2:** TaqMan assays used for qRT-PCR expression profiling.

Gene Symbol	Gene Name	Assay ID
*ZNF554*	Zinc finger protein 554	Hs00171072_m1
*IL6*	Interleukin 6	Hs00174131_m1
*IGFBP3*	Insulin-like growth factor-binding protein 3	Hs00181211_m1
*RPLP0*	Ribosomal protein lateral stalk subunit P0	Hs99999902_m1

## Supplementary Materials

The following are available online at https://www.mdpi.com/1422-0067/21/16/5762/s1.
