# Supplementary material for: Decreased Expression of ZNF554 in Gliomas is Associated with the Activation of Tumor Pathways and Shorter Patient Survival
Source: Int J Mol Sci. 2020 Aug 11;21(16):5762. doi: 10.3390/ijms21165762 (PMC7461028; doi:10.3390/ijms21165762)
Supplement: Supplementary file 1 [file ijms-21-05762-s001.zip › Supplementary Figure S1.docx]

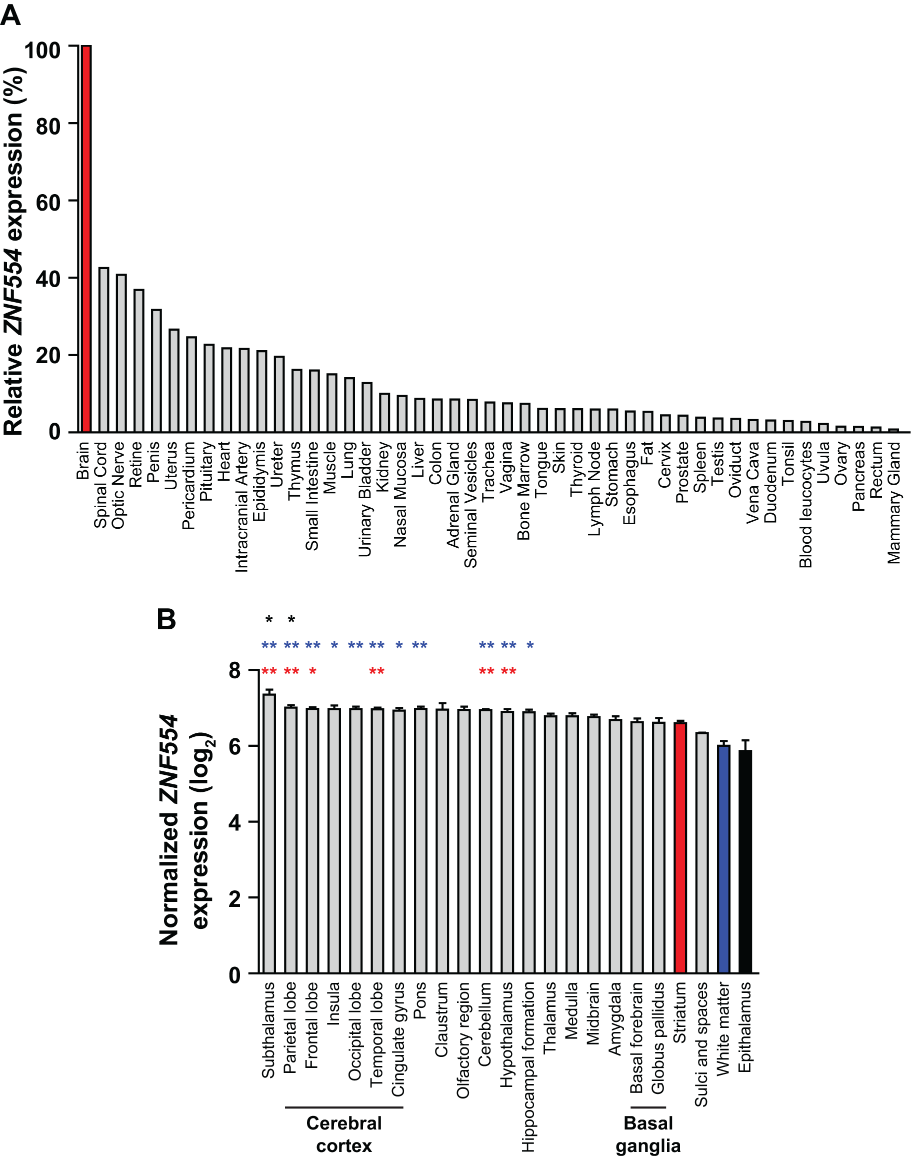


**Supplementary Figure S1. *ZNF554* mRNA expression profile in adult tissues and the neurotypical brain in healthy humans.** (**A**) The bar chart depicts normalized *ZNF554* gene expression levels, measured by qRT-PCR, in 47 human adult tissues relative to that of the brain (100%). (**B**) The Allen Brain Atlas was used to analyze *ZNF554* gene expression levels in human brain regions (n=6). The normalized microarray data (log_2_) are presented on a bar chart. Kruskal-Wallis with Dunn’s post hoc test was used to compare all groups (**p*<0.05, ***p*<0.01).
